# Sorafenib Ameliorates Renal Fibrosis through Inhibition of TGF-β-Induced Epithelial-Mesenchymal Transition

**DOI:** 10.1371/journal.pone.0117757

**Published:** 2015-02-13

**Authors:** Lining Jia, Xiaotao Ma, Baosong Gui, Heng Ge, Li Wang, Yan Ou, Lifang Tian, Zhao Chen, Zhaoyang Duan, Jin Han, Rongguo Fu

**Affiliations:** Department of Nephropathy, The Second Affiliated Hospital of Xi’an Jiaotong University, Xi’an, China; Faculty of Medicine &amp; Health Sciences, UNITED ARAB EMIRATES

## Abstract

**Objective:**

This study was to investigate whether sorafenib can inhibit the progression of renal fibrosis and to study the possible mechanisms of this effect.

**Methods:**

Eight-week-old rats were subjected to unilateral ureteral obstruction (UUO) and were intragastrically administered sorafenib, while control and sham groups were administered vehicle for 14 or 21 days. NRK-52E cells were treated with TGF-β1 and sorafenib for 24 or 48 hours. HE and Masson staining were used to visualize fibrosis of the renal tissue in each group. The expression of α-SMA and E-cadherin in kidney tissue and NRK-52E cells were performed using immunohistochemistry and immunofluorescence. The apoptosis rate of NRK-52E cells was determined by flow cytometry analysis. The protein levels of Smad3 and p-Smad3 in kidney tissue and NRK-52E cells were detected by western blot analysis.

**Results:**

HE staining demonstrated that kidney interstitial fibrosis, tubular atrophy, and inflammatory cell infiltration in the sorafenib-treated-UUO groups were significantly decreased compared with the vehicle-treated-UUO group (p<0.05). Masson staining showed that the area of fibrosis was significantly decreased in the sorafenib-treated-UUO groups compared with vehicle-treated-UUO group (p<0.01). The size of the kidney did not significantly increase; the cortex of the kidney was thicker and had a richer blood supply in the middle-dose sorafenib group compared with the vehicle-treated-UUO group (p<0.05). Compared with the vehicle-treated-UUO and TGF-β-stimulated NRK-52E groups, the expression of a-SMA and E-cadherin decreased and increased, respectively, in the UUO kidneys and NRK-52E cells of the sorafenib-treated groups (p<0.05). The apoptotic rate of NRK-52E cells treated with sorafenib decreased for 24 hours in a dose-dependent manner (p<0.05). Compared with the vehicle-treated UUO and TGF-β-stimulated NRK-52E groups, the ratio of p-Smad3 to Smad3 decreased in the sorafenib-treated groups (p<0.05).

**Conclusion:**

Our results suggest that sorafenib may useful for the treatment of renal fibrosis through the suppression of TGF-β/Smad3-induced EMT signaling.

## Introduction

Renal fibrosis is the final outcome of many chronic kidney diseases (CKDs) [1]. Activated myofibroblasts and epithelial-mesenchymal transition (EMT) play essential roles in the pathogenesis of renal fibrosis [2]. However, the regulatory mechanisms of renal fibrosis processes are not fully understood, and there is currently no effective treatment.

There is increasing evidence that the transforming growth factor (TGF)-β pathway is a potent moderator of progressive renal fibrosis. EMT is also known to be involved in various physiological and pathological states, including organ fibrosis [3]. TGF-β triggers EMT primarily via a Smad-dependent mechanism [4]. Smad2/3 is phosphorylated by Smad4 and then translocates to the nucleus, where it regulates the transcription of the target genes responsible for EMT [5]. Therefore, we hypothesized that inhibiting the TGF-β/Smad pathway would slow or reverse the process of renal fibrosis.

Sorafenib, a multi-kinase inhibitor, was initially approved for use in humans with renal cancer and liver cancer [6]. Sorafenib significantly inhibits epithelial cancer cell proliferation and EMT [7]. Furthermore, sorafenib is known to target both Raf and several tyrosine kinases, including vascular endothelial growth factor R2 (VEGF-R2), platelet-derived growth factor (PDGF) receptor, and VEGF receptor [8], and to regulate receptor tyrosine kinase pathways in adjacent stromal cells, including myofibroblasts and endothelial cells [9]. Interestingly, myofibroblast activation and endothelial cell proliferation contribute to matrix production and vascular sclerosis during renal fibrosis. In addition, previous studies have demonstrated that sorafenib has potential action in the treatment of liver and lung fibrosis [10, 11, 12, 13]. Thus, sorafenib may be able to ameliorate renal fibrosis through inhibition of TGF-β-induced EMT. This study aimed to explore the therapeutic potential and possible mechanisms of action of sorafenib in renal fibrosis. Therefore, we examined the effects of sorafenib on TGF-β-mediated EMT in NRK-52E cells in vitro and in a rat model of UUO renal fibrosis.

## Materials and Methods

### Reagents and Antibodies

Sorafenib (Nexavar, BAY43-9006) is manufactured by Bayer Pharmaceuticals (West Haven, CT, USA). Recombinant human TGF-β1 was purchased from R&D Systems (Minneapolis, MN, USA), and primary antibodies against Smad3 and p-Smad3 were purchased from Cell Signaling Technology (Beverly, MA, USA). The rabbit monoclonal antibodies against a-SMA and E-cadherin were purchased from Sigma-Aldrich (St. Louis, MO, USA), and the monoclonal anti-β-actin antibody was purchased from Sigma Chemical Company (St. Louis, MO, USA).

### Establishment of the UUO Model

Forty male Sprague-Dawley rats were used in this study and were given free access to water and food throughout the experiments. The rats were acclimatized for at least 1 week prior to the experiments. The UUO model was established in groups of eight rats (males, 8 weeks of age, 162 to 202 g body weight) by left ureteral ligation. Eight normal sham rats were used as control group.

### Experimental Protocol

The rats were divided into 5 groups (eight rats per group). The experimental rats were administered sorafenib intragastrically (20, 40, 80 mg/kg·d), and the UUO and sham group rats were administered vehicle for 14 or 21 days. Kidney tissue samples were collected for HE and Masson staining. The protein expression of a-SMA and E-cadherin in kidney tissue was assessed by immunohistochemistry and immunochemistry. The expression of Smad3 and phosphorylated Smad3 in kidney tissue was detected by western blot analysis. All of the experimental protocols described in this study were approved by the local committee for animal use and care (Animal Care Committee of Xi'an Jiaotong University).

### Cell Culture

NRK-52E cells were cultured in Dulbecco’s modified Eagle’s medium (DMEM) and 10% fetal bovine serum at 37°C in 5% CO_2_. The cells were divided into the following six groups: control group, TGF-β (5 ng/mL) group, sorafenib group (10 umol/L), and TGF-β (5 ng/mL) co-treated with sorafenib (1, 5 and 10 umol/L) groups. NRK-52E cells were treated in vitro with TGF-β1 and sorafenib for 24 or 48 hours.

### HE staining, Masson staining, Immunohistochemistry, and Immunofluorescence of Kidney Tissue

The tissue was stained with hematoxylin, and ponceau red liquid dye acid complex was then administered, followed by soaking in 1% phosphomolybdic acid solution. The tissue was then directly stained with aniline blue liquid and 1% acetic acid, followed by dehydration with a series of differing ethanol concentrations. Staining showed collagen fibers in blue and cytoplasm in red.

The renal tissue sections were fixed for 10 min in acetone and immersed in 0.3% H_2_O_2_ for 30 min to quench endogenous peroxidase activity. The sections were incubated at room temperature with an optimal dilution of a-SMA and E-cadherin monoclonal antibodies for 1 hour. After the sections were washed with phosphate-buffered saline (PBS), anti-rabbit secondary antibody was added for 30 min. The sections were then washed with PBS, incubated with an ABC kit for 30 min, developed with 3,3’-diaminobenzidine (DAB), and counterstained with hematoxylin. The IPP6 was assessed to quantify the expression of a-SMA and E-cadherin in the kidney tissue.

HE and Masson staining as well as immunostaining intensity were scored, and the scoring criteria were as follows: 10 high-power fields (x200) were randomly selected and photographed in each group. None, mild, moderate and severe involvement were scored as 0, 1, 2, or 3 according to the degree and extent of tubular degeneration and necrosis, tubular atrophy, inflammatory cell infiltration and fibrosis. The blue area of collagen by Masson staining, which represents the extent of the lesion, was calculated. The dyed area was measured to calculate the average optical density in the immunostaining intensity scores.

### Western Blot Analysis

After proteins were isolated from the kidney tissues or cells, proteins were transferred to membranes, and the membranes were incubated overnight at 4°C with rabbit anti-rat Smad3 and p-Smad3 monoclonal antibodies. After the membranes were washed repeatedly with Tris buffer containing 0.1% Tween-20 (TBST), they were incubated with secondary antibodies. The blots were assessed by the enhanced chemiluminescence method. The Smad3 and p-Smad3 levels were normalized to β-actin as an internal control. The expression of Smad3 and phosphorylated Smad3 in NRK-52E cells and kidney tissue was assessed by western blot analysis.

### Flow Cytometry Analysis

NRK-52E cells from each group were collected and stained with FITC-labeled Annexin V and propidium iodide (PI). The apoptosis rate of NRK-52E cells was determined by flow cytometry analysis (FCM; Becton Dickinson, San Jose, CA).

### Statistical Analysis

Statistical analysis was performed using SPSS software, version 19.0. The data are expressed as the means ± standard deviations (SDs). The statistical significance of differences was calculated using the t-test and one-way analysis of variance (ANOVA), and p≤0.05 was considered statistically significant.

## Results

### Sorafenib Attenuates Kidney Tissue Injury in UUO

**Fig 1:**
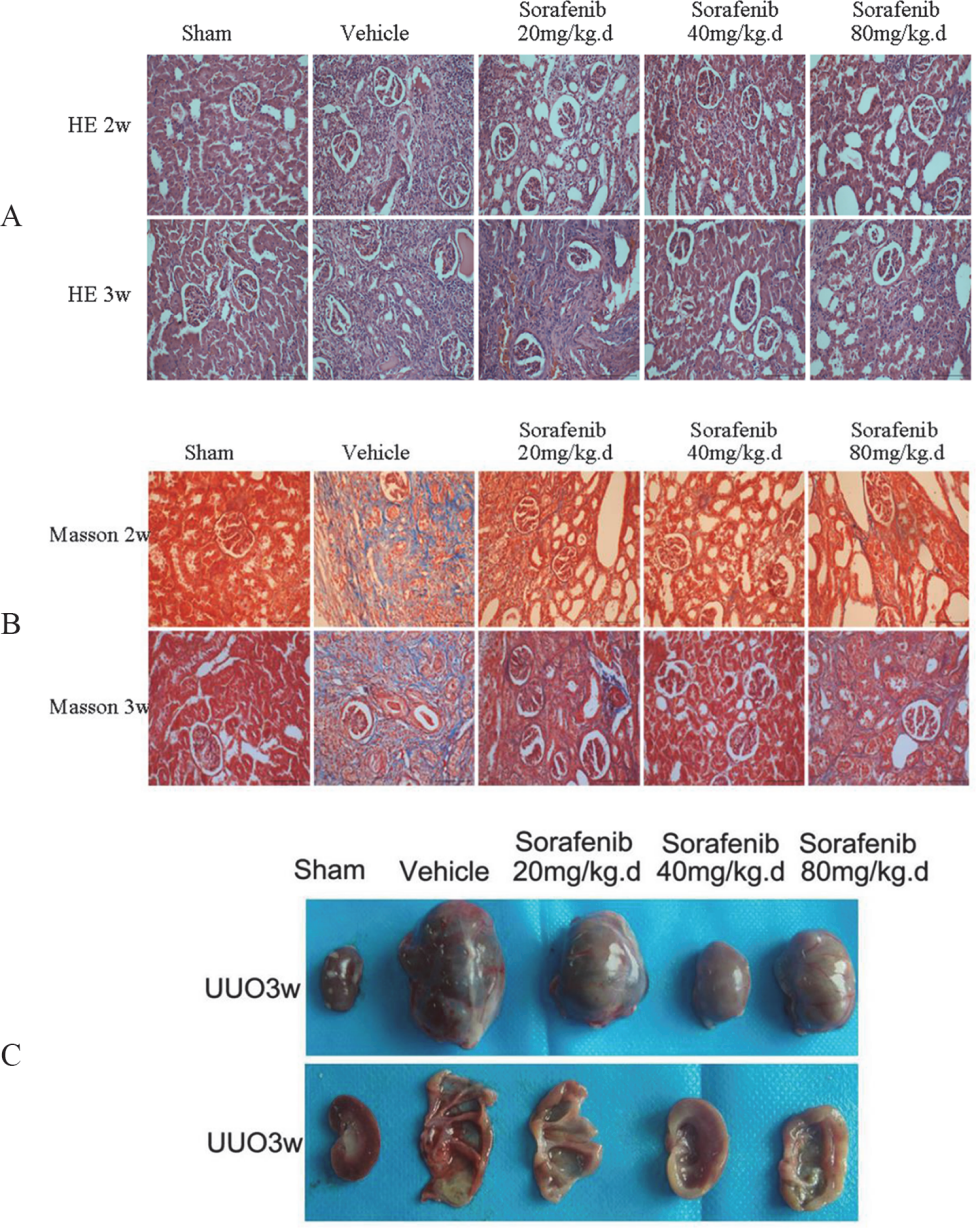
HE, Masson staining of UUO kidney tissue. (A) HE staining demonstrated that compared with the vehicle-treated-UUO, kidney interstitial fibrosis, tubular atrophy, and inflammatory cell infiltration were decreased in the sorafenib-treated-UUO groups (*p*<0.05), especially in the medium-dose sorafenib group (*p*<0.01). (B) The results of Masson staining showed that there was less collagen deposition around the renal tubules and that the area of fibrosis in the UUO group treated with the middle dose of sorafenib was significantly decreased compared with the vehicle-treated-UUO group and sham group (*p*<0.01). (C) The size of the kidney was not significantly increased and the cortex of the kidney was thicker and had a richer blood supply in the sorafenib middle-dose group compared with the control group (*p*<0.05).

HE staining demonstrated that compared with the UUO group, renal interstitial fibrosis, tubular atrophy, and inflammatory cell infiltration were significantly decreased in the UUO group treated with sorafenib (*p*<0.05), especially the medium-dose sorafenib group (Fig. 1A, *p*<0.01). The results of Masson staining showed that there was less collagen deposition around the renal tubules and that the area of fibrosis in the sorafenib-treated-UUO groups was significantly decreased compared with the vehicle-treated-UUO group (Fig. 1B, *p*<0.01). In addition, in the group treated with the middle dose of sorafenib, the size of the kidney was not significantly increased; the cortex of the kidney was thicker and had a richer blood supply compared with the vehicle-treated-UUO group (Fig. 1C, *p*<0.05).

### Sorafenib Inhibits Kidney Tissue Fibrosis

**Fig 2:**
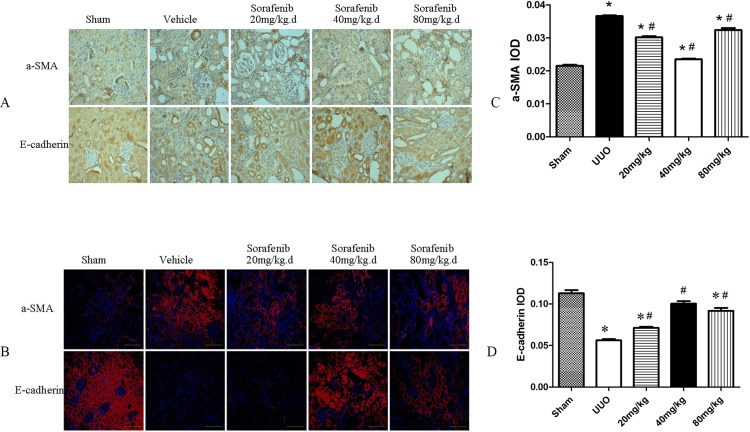
Immunohistochemical and immunofluorescence staining of E-cadherin and a-SMA protein in kidney tissue. (A-D) Immunohistochemistry and immunofluorescence demonstrated that E-cadherin was expressed in the nuclei and interstitium. Compared with the vehicle-treated-UUO group, the expression of a-SMA and E-cadherin decreased and increased, respectively, in the UUO kidneys of sorafenib-treated groups (*p*<0.05).

Immunohistochemistry and immunofluorescence demonstrated that E-cadherin was mainly expressed in the nuclei and interstitium. The expression of a-SMA and E-cadherin increased and decreased, respectively, in the kidneys of the UUO group compared with the sham group. These changes were partly reversed after treatment with sorafenib. These changes were most obvious in the group treated with the middle dose (*p*<0.05, Fig. 2).

### Sorafenib Inhibits TGF-β-induced EMT and Apoptosis in NRK-52E Cells

**Fig 3:**
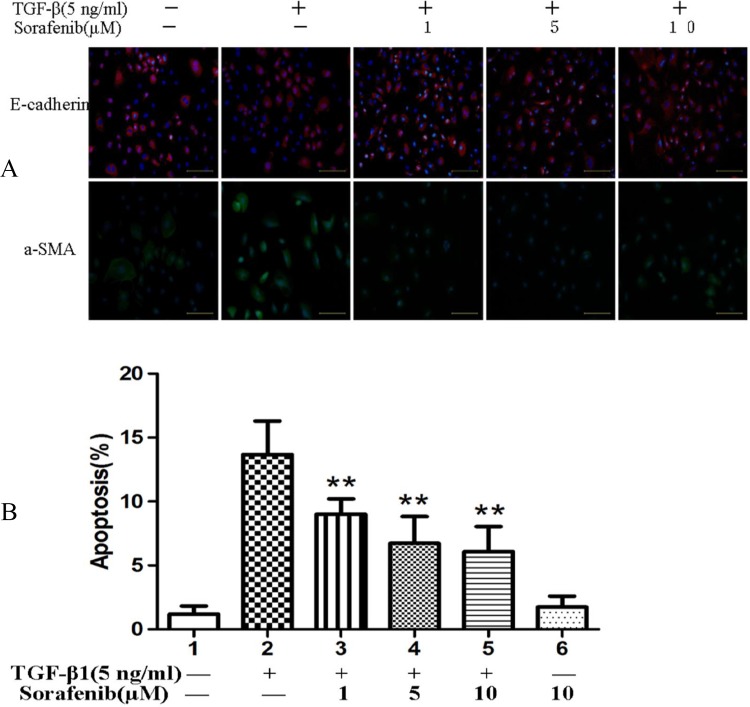
Immunofluorescence staining of E-cadherin and a-SMA protein and analysis of apoptosis in NRK-52E cells. (A) The expression of E-cadherin was increased, and the expression of a-SMA was decreased in the sorafenib-treated group relative to the TGF-β1-stimulated group (*p*<0.05). (B) The apoptotic rates were measured by flow cytometry. The rate of apoptosis in the NRK-52E cells began to significantly increase after 24 h of TGF-β1 stimulation. Sorafenib effectively reduced apoptosis in the TGF-β1-stimulated groups (*p*<0.05).

To assess whether sorafenib inhibits TGF-β-induced EMT in renal tubule epithelial cells, we used rat NRK-52E cells as an in vitro model system for assessing EMT. E-cadherin (red) and a-SMA (red) were expressed in the cytoplasm of NRK-52E cells. Compared with TGF-β1 stimulation, E-cadherin and a-SMA expression were increased and decreased, respectively, in the sorafenib treatment groups, especially in the group receiving the 5 μM sorafenib dose (*p*<0.05, Fig. 3A). The apoptotic rates were measured by flow cytometry analysis. The apoptotic rate of NRK-52E cells treated with sorafenib was decreased for 24 hours in a dose-dependent manner (*p*<0.05, Fig. 3B).

### Sorafenib Inhibits the Expression of Smad3 and Phosphorylated Smad3 in Kidney Tissue and NRK-52E Cells

**Fig 4:**
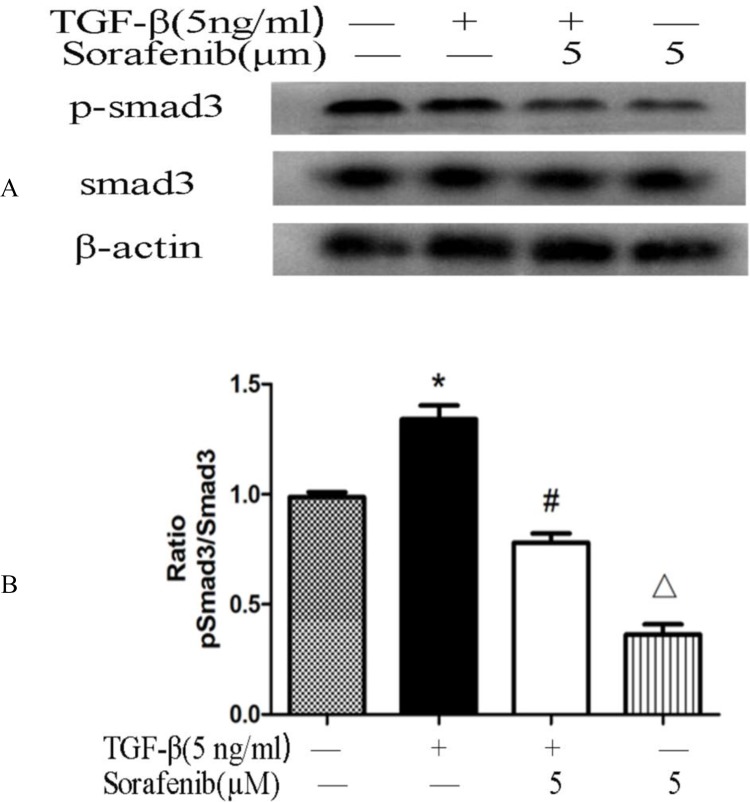
Expression of Smad3 and phosphorylated Smad3 in NRK-52E cells. (A, B) Western blotting was performed to detect the protein levels of p-Smad3 and Smad3, and beta-actin was used as a loading control. The ratio of p-Smad3 to Smad3 was decreased in the sorafenib-treated groups relative to the TGF-β1-stimulated group in a dose-dependent manner (*p*<0.05).

**Fig 5:**
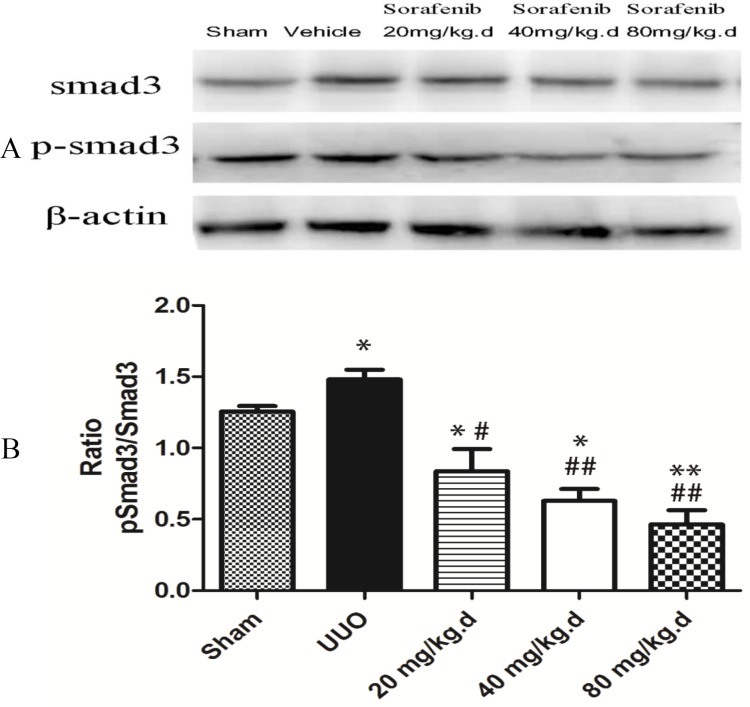
Expression of Smad3 and phosphorylated Smad3 in kidney tissue. (A, B) Sorafenib-treated groups compared with the vehicle-treated UUO group; the ratio of p-Smad3 to Smad3 was decreased in a dose-dependent manner (*p*<0.05).

Western blotting was performed to assess the protein levels of p-Smad3 and Smad3 in kidney tissue and NRK-52E cells, β-actin was used as a loading control. Compared with the vehicle-treated UUO and TGF-β1-stimulated NRK-52E cells groups, the ratio of p-Smad3 to Smad3 was decreased in the sorafenib treatment groups (*p*<0.05, Figs. 4 and 5).

## Discussion

The present study demonstrated that sorafenib ameliorated kidney fibrosis in a UUO model and inhibited EMT and apoptosis induced by TGF-β in NRK-52E cells in vitro. Our data are consistent with those of Chen et al. [12, 13], who found that sorafenib inhibits TGF-β-induced EMT and apoptosis in mouse hepatocytes. In recent years, an increasing number of studies have examined kinase inhibitor amelioration of organ fibrosis [14, 15, 16]. Zhang et al. [17] proposed that sorafenib inhibits both TGF-β1-induced EMT through a possible epigenetic mechanism and the subsequent epigenetic switching of relevant genes that are critical for EMT in human lung epithelial cells. Jin et al. [18] found that knockout of homeodomain-interacting protein kinase 2 (HIPK2) improves renal function and attenuates proteinuria and kidney fibrosis in animal models of kidney fibrosis. Recent studies have also suggested that serine protease inhibitors may provide a new class of therapeutic drugs for the treatment of renal fibrosis through the suppression of TGF-β signaling [19, 20, 21]. Consistent with these studies, we demonstrated that sorafenib reduced the rate of apoptosis and EMT in TGF-β-induced NRK-52E cells and ameliorated renal fibrosis in a UUO model, suggesting a potential and novel use of the drug in the treatment of renal fibrosis.

In 2005, sorafenib became the first FDA-approved oral agent for the treatment of patients with advanced hepatocellular and renal cell carcinomas [6]. Previous reports largely focused on the role of sorafenib in tumors and apoptosis via blocking receptor tyrosine kinases. In this study, we discovered a novel action of sorafenib, namely, it significantly suppressed TGF-β-induced EMT and apoptosis in NRK-52E cells and attenuated renal fibrosis in a UUO model. The available evidence has shown that TGF-β is the major mediator of progressive renal fibrosis, largely via the Smad-dependent pathway. However, the exact molecular mechanisms underlying the link between TGF-β and disease progression in kidney fibrosis have remained elusive. These findings provide the first evidence that sorafenib ameliorates renal fibrosis through a TGF-β-mediated, Smad-dependent mechanism [22].

However, several limitations of our study must be acknowledged. This investigation was primarily limited to the TGF-β/Smad pathway; other detailed underlying molecular mechanisms should be explored in future studies. In addition, the details of how sorafenib inhibits kidney fibrosis should also be further explored in future studies. In summary, we found that sorafenib is a potential therapeutic agent for the treatment of kidney fibrosis through inhibition of the TGF-β/Smad pathway.
